# Strengthening the community health worker programme for health improvement through enhancing training, supervision and motivation in Wakiso district, Uganda

**DOI:** 10.1186/s13104-019-4851-6

**Published:** 2019-12-18

**Authors:** David Musoke, Charles Ssemugabo, Rawlance Ndejjo, Edwinah Atusingwize, Trasias Mukama, Linda Gibson

**Affiliations:** 1 Department of Disease Control and Environmental Health, School of Public Health, College of Health Sciences, Makerere University, Kampala, Uganda; 2 School of Social Sciences, Nottingham Trent University, Nottingham, UK

**Keywords:** Community health workers, Village health teams, Training, Supervision, Motivation, Performance, Uganda

## Abstract

**Objective:**

The objective of the project was to strengthen the community health worker (CHW) programme in Ssisa sub-county, Wakiso district, Uganda by providing a coherent, structured and standardized training, supervision and motivation package so as to enhance their performance.

**Results:**

The project trained all 301 CHWs who received non-financial incentives of t-shirts, gumboots and umbrellas, and 75 of them received solar equipment to support lighting their houses and charging phones. Twenty-four of the CHWs who had coordination roles received additional training. Three motorcycles were also provided to enhance transportation of CHW coordinators during their work including supervision. By end of the project, the CHWs had conducted 40,213 household visits, carried out health education sessions with 127,011 community members, and treated 19,387 children under 5 years of age. From the project evaluation, which used both quantitative and qualitative methods, 98% of the CHWs reported having improved competence in performance of their roles. In addition, the CHWs were highly motivated to do their work. The motorcycles were instrumental in supporting the work of CHW coordinators including monthly collection of reports and distribution of medicines. The project demonstrated that by improving training, supervision and motivation, performance of CHW programmes can be enhanced.

## Introduction

Community health workers (CHWs) play a major role in prevention and control of diseases in many low- and middle-income countries (LMICs) [1–3] including Uganda [4–8]. Due to constraints in human resources for health, Uganda established a CHW programme in 2001 through the National Health Policy of 1999 as part of the Uganda National Minimum Health Care Package (UNMHCP). The aim of the UNMHCP was to ensure all villages in the country have the capacity to mobilise individuals and households for better health outcomes [9–11]. CHWs in Uganda (locally referred to as Village Health Teams (VHTs)] are volunteers selected from their communities as the first link with the health system. The roles of CHWs include community mobilisation, health promotion at household and community levels, and linking the population with health facilities including referral of patients. Where CHWs are functional, they have contributed to: raising health awareness; increased demand and utilisation of health services; and decongestion at health facilities as they treat minor childhood illnesses of malaria, diarrhoea and pneumonia. CHWs have further helped to increase community participation in local health programmes in Uganda [12].

Despite evidence that CHWs promote adoption of healthy behaviours and access to several services, challenges exist that affect their programmes as well as level of performance and retention. These challenges include: high attrition levels of up to 77% where CHWs are volunteers [13], and poor performance for those who stay in job [14]. A situation analysis of CHWs conducted by the Ministry of Health (MOH), Uganda found major shortfalls in the areas of training, supervision and motivation among other issues [15] which still remain a challenge [16]. Moreover, support supervision, recognition, training, and availability of equipment and supplies have been identified to be critical elements that improve CHWs’ level of activity and retention [13]. Therefore, exploring ways of improving and sustaining the CHW programme is a priority for MOH [17] as part of efforts to attain health for all. This project therefore sought to strengthen the CHW programme in Ssisa sub-county, Wakiso district, Uganda by providing a coherent, structured and standardized training, supervision and motivation package so as to enhance the performance of CHWs. This paper presents details of project implementation including findings from monitoring and the final evaluation.

## Main text

### Implementing partners

The project was implemented by the partnership between Nottingham Trent University (NTU), UK and Makerere University School of Public Health (MakSPH), Uganda. Other local implementing partners were MOH and Wakiso District Local Government.

### Project design and context

The project was implemented over a period of two and a half years (2014 to 2017) in Ssisa sub-county. The sub-county had a population of 94,238 [18] and is located in Wakiso district in the central region of Uganda. Wakiso district had the largest population (2,007,700) in the country [18], and neighbours Kampala, Uganda’s capital city. The project involved all CHWs in the sub-county whether they were involved in integrated community case management of childhood illnesses (iCCM) or not. All CHW parish coordinators in the sub-county, who offered assistance to fellow CHWs in their areas of jurisdiction, received additional support in the project.

### Project implementation

#### Preparatory work

Several planning meetings among the project team partners were held which were critical in engaging various stakeholders, creating awareness, and laying a foundation for project ownership and sustainability. The project had three phases of baseline, intervention and evaluation. The baseline phase comprised of a survey to establish status of the CHW programme in the area and aid planning for project implementation. The intervention phase of the project was based on enhancing training, supervision and motivation. These three aspects of the CHW programme were identified through a needs assessment carried out earlier in the project area.

#### Training

The project trained 301 CHWs of whom 24 were coordinators who received additional training. The CHWs’ training covered a range of topics including: water, sanitation and hygiene; communicable and non-communicable diseases; maternal and child health; communication; and reporting. There was an initial 4-day training in year 1 with the first 2 days for all CHWs, while the final 2 days were specifically for those involved in iCCM. In year 2 of the project, a 1-day refresher training for all CHWs was held. An additional 1-day training for CHW coordinators covered their responsibilities, communication, record keeping and reporting. All trainings were conducted by health practitioners from health facilities in the area. The venue for the trainings were available sites in the community including schools and churches.

#### Supervision

The project supported the CHW coordinators with three motorcycles that were used to collect reports from CHWs as well as provide other supervisory support including distribution of medicines and other supplies.

#### Motivation

**Fig. 1:**
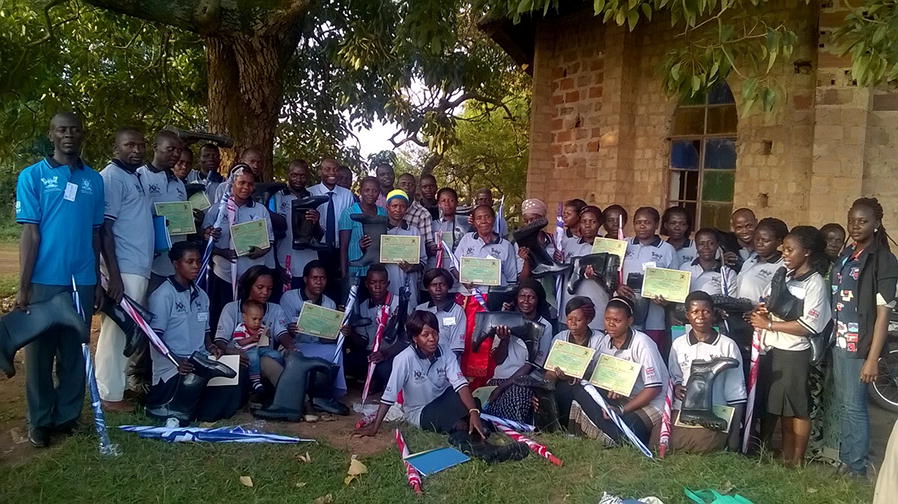
A group of community health workers in their t-shirts display certificates, umbrellas and gum boots received after a project training

Motivation was enhanced by providing non-financial incentives of t-shirts, umbrellas, gum boots and certificates to all CHWs (Fig. 1), and solar equipment to 75 of them. Monthly mobile telephone credit was provided to coordinators for improved communication with their respective CHWs. Among the CHWs trained, 236 (78%) were females, and 136 (45%) involved in iCCM (Table 1). More details on the needs assessment, results of the baseline survey as well as the implementation phase of the project are in our earlier publications [19, 20]. This paper has therefore focused more on project monitoring and final evaluation.

**Table 1:** Numbers of community health workers trained by gender and iCCM involvement

Month (2015/16)	Total	Gender		CHW roles	
		Male	Female	iCCM	Non-iCCM
September	66	16	50	30	36
October	60	9	51	28	32
November	74	22	52	32	42
December	73	17	56	32	41
January	28	1	27	14	14
Total	301	65 (22%)	236 (78%)	136 (45%)	165 (55%)

### Project monitoring

**Fig. 2:**
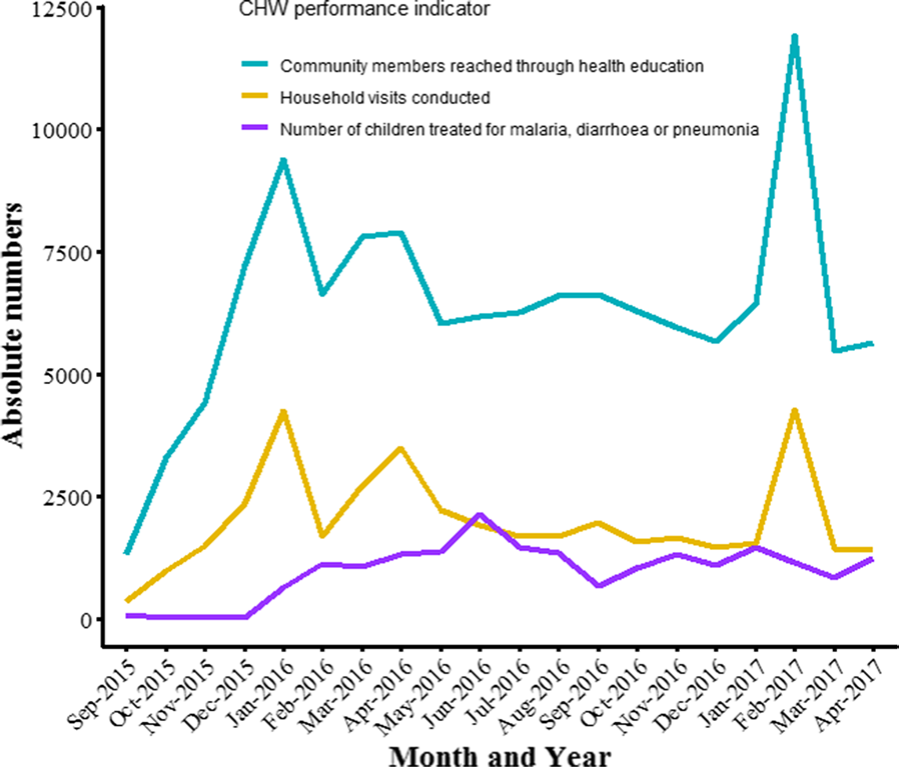
Summary of community health workers’ performance on key indicators over the project period

Project monitoring involved routine collection of data by CHWs to establish their performance based on stipulated roles and responsibilities. Completed monitoring data reporting forms were collected from CHWs every month by their coordinators. For all CHWs, performance indicators monitored by the project were: number of household visits conducted, and number of community members reached through health education. For CHWs involved in iCCM, the number of children under 5 years of age treated for malaria, diarrhoea or pneumonia were an additional indicator monitored monthly. The training of CHWs was done incrementally, and collection of monitoring data was commenced the month after their respective training. Given the final main training of CHWs was conducted in January 2016, collection of monitoring data for the entire 301 CHWs commenced the following month (Fig. 2). The sharp decline in performance of CHWs regarding health education and household visits in February 2016 was due to Presidential and Parliamentary elections held in Uganda that month. Otherwise, there was a general slight decline in CHW performance for these two indicators in the proceeding months till February 2017 when there was a nationwide health campaign in which CHWs were heavily involved. By the end of the project, CHWs had conducted 40,213 household visits; carried out health education among 127,011 community members (56,415 among males and 70,596 females); and treated 19,387 children suffering from malaria, diarrhoea or pneumonia.

### Project evaluation

#### Methodology

The objective of the end-term project evaluation was to assess the: performance of CHWs including their competence to do designated roles; motivation of CHWs; and the level of support CHWs received from their coordinators. In addition, the evaluation explored perspectives of various stakeholders (community, health practitioners and local leaders) about CHWs and their performance following the project’s interventions. In the evaluation, both quantitative and qualitative data were collected. Questionnaires were used to collect quantitative data from all CHWs including coordinators who all accepted to take part. The questionnaires mainly assessed performance of the CHWs in relation to their competence to carry out their roles, motivation following receiving non-financial incentives, and supervision from their coordinators. Focus group discussions (FGDs) were employed to collect qualitative data from CHWs, their coordinators and community members. Key informant interviews (KIIs) were conducted among health practitioners from health facilities in the area, district health officials, and local leaders. A total of six FGDs were carried out (two for CHWs, three for community members, and one for coordinators), and six key informants were interviewed. Each FGD comprised of between 6 and 12 participants, and were facilitated by trained Research Assistants. Univariate analysis was carried out for the quantitative data while thematic analysis was employed for qualitative data.

### Results

#### Improved performance of CHWs

A total of 298 (99%) CHWs were reached in the project evaluation. Overall, high performance of CHWs was reported as the majority 292 (98%) felt competent in their roles including diagnosis, record keeping and referral of sick children. Ongoing training sessions conducted among CHWs, as were carried out in the project, have been shown to be necessary to support their work in LMICs [21]. The performance of CHWs following the project’s interventions was also rated highly by key informants as well as community members.

*“The skills of CHWs in our village have improved a lot as they cannot treat children suffering from malaria without testing them first. The trainings offered to them have been of great help. On the other hand, when we visit some private clinics, they just treat our children without first testing them.”* Community member

#### Enhanced motivation of CHWs

The evaluation indicated that the CHWs including their coordinators were highly motivated to carry out their roles and responsibilities. The CHWs reported being satisfied with the non-financial incentives they received such as solar equipment which enabled them to charge their phones at home besides providing lighting to be able to work at night. The gumboots and umbrellas motivated CHWs to confidently conduct home visits even during difficult weather conditions. For example, CHWs were able to carry out community work during periods of heavy rain thus improving general performance. Non-financial incentives have been shown to greatly motivate CHWs in many countries [22] so should be provided routinely especially in voluntary programmes such as in Uganda. Branded t-shirts provided by the project were also important for identity and status of the CHWs.

*“In the past, people used to call us to treat their sick children when it was raining yet we had no gumboots hence could not help them. However, when the project commenced, we were given gumboots and umbrellas to do our work even in bad weather conditions such as when it rains. In addition, whenever I move in my village dressed in my t*-*shirt, everyone knows that I am a CHW which makes me easily identifiable hence my work becomes easier.”* Community health worker

#### Improved supervision of CHWs

Improved supervision of CHWs and support from their coordinators greatly enhanced performance of their roles especially household visiting, health education and treatment of childhood illnesses. Results of the evaluation also revealed that most CHWs were satisfied with the supervisory support they received from their coordinators. The motorcycles provided by the project particularly made it easy for coordinators to provide the necessary support to CHWs. As many coordinators have to cover long distances to reach CHWs [23] and in difficult terrain in rural areas, such transportation is of paramount importance. Regular phone calls and visits made by coordinators to the CHWs were also reported to be beneficial.

*“Our coordinators communicate with us frequently including calling us before collecting monthly reports. They also notify us whenever medicines and other supplies have been availed from the health facility. In case of any important information, they also call or visit us to let us know. This ensures we are up to date with what is going on regarding our work, and we really thank them for this support.”* Community health worker

### Conclusion

The project demonstrated enhanced performance of CHWs through supporting: coherent, structured and standardized training; supervision through enhanced transportation; and motivation through non-financial incentives. Lessons from this project can inform interventions in other parts of the country as well as national policy decisions to improve the CHW programme.

## Limitations

A limitation of the study was that the performance data from CHWs was self-reported which could have introduced some bias. However to minimise this bias, the CHWs were informed of the importance of being truthful in their responses to positively inform the interventions of the project. In addition, other health systems challenges such as occasional stock out of medicines may have affected performance of the CHWs especially regarding number of children treated.

## Data Availability

Data and materials of the project are available from the corresponding author on reasonable request.

## Abbreviations

CHW: community health worker
FGD: focus group discussion
iCCM: integrated community case management of childhood illnesses
KII: key informant interview
LMIC: low- and middle-income country
MOH: Ministry of Health
